# G6PD and chloroquine: Selecting the treatment against SARS‐CoV‐2?

**DOI:** 10.1111/jcmm.15312

**Published:** 2020-04-16

**Authors:** Eva N. Kassi, Kostas A. Papavassiliou, Athanasios G. Papavassiliou

**Affiliations:** ^1^ Department of Biological Chemistry Medical School National and Kapodistrian University of Athens Athens Greece

**Keywords:** chloroquine, COVID‐19 (SARS‐CoV‐2), G6PD, haemolysis, oxidative injury, pandemic, retinopathy

In light of the severe acute respiratory syndrome coronavirus 2 (SARS‐CoV‐2 or COVID‐19) pandemic and the possible widespread use of chloroquine (a member of the drug class 4‐aminoquinoline primarily used to prevent and treat malaria and amoebiasis) and its derivatives (eg hydroxychloroquine, a metabolite of chloroquine),^1^ a safety issue is addressed, concerning the selection of patients suitable to receive it. HCov‐229E is one of the four flu‐causing coronaviruses which share great sequence similitude, while many symptoms of patients infected with COVID‐19 resemble those of patients infected with these known flu‐causing coronaviruses.^2^ A previous study reported that human lung epithelial A549 cells treated with glucose 6‐phosphate dehydrogenase (G6PD) interfering RNA (RNAi) to lower G6PD activity displayed augmented (12‐fold) viral production in comparison with normal counterparts when infected with coronavirus HCov‐229E. Moreover, viral replication in these G6PD‐deficient cells was found to be 3‐fold higher than normal cells, following a 10‐hour incubation, as estimated by quantitative polymerase chain reaction (qPCR).^3^ G6PD deficiency remains the most common human enzymatic disorder of red blood cells worldwide. It is an X‐linked disorder affecting about 400 million people with a higher prevalence across African, Asian, Latin America, and Mediterranean countries.^4^ G6PD is known to catalyse the initial step in the pentose phosphate pathway. The main function of this pathway, also known as hexose monophosphate shunt, is to protect red blood cells against oxidative stress through the production of nicotinamide adenine dinucleotide phosphate (NADPH) which is generated by G6PD.^5^ It is well established that chloroquine‐induced haemolysis is a result of reduced G6PD enzyme activity. The increased oxidative injury caused by chloroquine has long been thought to be responsible for the drug's toxicity mainly leading to retinopathy (blurred and partial loss of central vision, side vision and in the later stage, night vision).^6^ Taking into account that G6PD‐deficient subjects may be more susceptible to human coronavirus (a new predisposing factor?) and as such more plausible candidates to receive chloroquine or its analogs, physicians may need to be more careful when treating COVID‐19 patients with chloroquine and its derivatives, especially at large doses. Since experts predict that COVID‐19 will spread more widely in countries with higher prevalence of G6PD deficiency, treatment decisions may merit serious consideration and need to be properly adapted.

## CONFLICT OF INTEREST

The authors confirm that there are no conflicts of interest.
